# Retraction Note: Modified box dimension and average weighted receiving time on the weighted fractal networks

**DOI:** 10.1038/s41598-020-63244-9

**Published:** 2020-04-08

**Authors:** Meifeng Dai, Yanqiu Sun, Shuxiang Shao, Lifeng Xi, Weiyi Su

**Affiliations:** 1 Nonlinear Scientific Research Center, Faculty of Science, Jiangsu University Zhenjiang, Jiangsu, 212013 P.R. China; 2 Institute of Mathematics, Zhejiang Wanli University, Ningbo, 315100 P.R. China; 3 Department of Mathematics, Nanjing University, Nanjing, 210093 P. R. China

Retraction of: *Scientific Reports* 10.1038/srep18210, published online 15 December 2015

The Editors have retracted this Article because significant portions of the text and equations were taken from Roland Molontay’s BSc thesis without attribution^1^.

The following parts of the paper are copied verbatim or are adapted from those appearing in the thesis: the definition of dB and preceding and subsequent sentences; Definition 3.2; Proof of Lemma 3.4; Lemma 3.5; Proof of Lemma 3.5; Lemma 3.6; Proof of Lemma 3.6; Equation 6; Lemma 3.7; Proof of Lemma 3.7; Lemma 3.8; Equation 8; Proof of Theorem 3.3.

The authors do not agree to the retraction of the Article.
